# Exploring ionic liquid-laden metal-organic framework composite materials as hybrid electrolytes in metal (ion) batteries

**DOI:** 10.3389/fchem.2022.995063

**Published:** 2022-09-14

**Authors:** Maitane Urgoiti-Rodriguez, Saloa Vaquero-Vílchez, Alexander Mirandona-Olaeta, Roberto Fernández de Luis, Eider Goikolea, Carlos M. Costa, Senentxu Lanceros-Mendez, Arkaitz Fidalgo-Marijuan, Idoia Ruiz de Larramendi

**Affiliations:** ^1^ Departamento de Química Orgánica e Inorgánica, Facultad de Ciencia y Tecnología, Universidad del País Vasco/Euskal Herriko Unibertsitatea (UPV/EHU), Leioa, Spain; ^2^ BCMaterials, Basque Center for Materials, Applications and Nanostructures, UPV/EHU Science Park, Leioa, Spain; ^3^ Physics Centre of Minho and Porto Universities (CF-UM-UP), University of Minho, Braga, Portugal; ^4^ Institute of Science and Innovation for Bio-Sustainability (IB-S), University of Minho, Braga, Portugal; ^5^ Laboratory of Physics for Materials and Emergent Technologies, LapMET, University of Minho, Braga, Portugal; ^6^ Ikerbasque, Basque Foundation for Science, Bilbao, Spain

**Keywords:** metal-organic frameworks, ionic liquid, electrolyte, battery, lithium, sodium

## Abstract

This review focuses on the combination of metal-organic frameworks (MOFs) and ionic liquids (ILs) to obtain composite materials to be used as solid electrolytes in metal-ion battery applications. Benefiting from the controllable chemical composition, tunable pore structure and surface functionality, MOFs offer great opportunities for synthesizing high-performance electrolytes. Moreover, the encapsulation of ILs into porous materials can provide environmentally benign solid-state electrolytes for electrochemical devices. Due to the versatility of MOF-based materials, in this review we also explore their use as anodes and cathodes in Li- and Na-ion batteries. Finally, solid IL@MOF electrolytes and their implementation into Li and Na batteries have been analyzed, as well as the design and advanced manufacturing of solid IL@MOF electrolytes embedded on polymeric matrices.

## Introduction

Nowadays, climate change and its effects on the environment is one of the main problems affecting humanity. This phenomenon has been occurring since the early history of the Earth but due to natural causes. However, in recent decades the increase of greenhouse gas (GHG) emissions have accelerated the global warming. The principal source of emission of GHGs are of anthropogenic origin such as combustions, industrial processes and transport, being the main source of emissions the combustion of fossil fuels.

**FIGURE 1:**
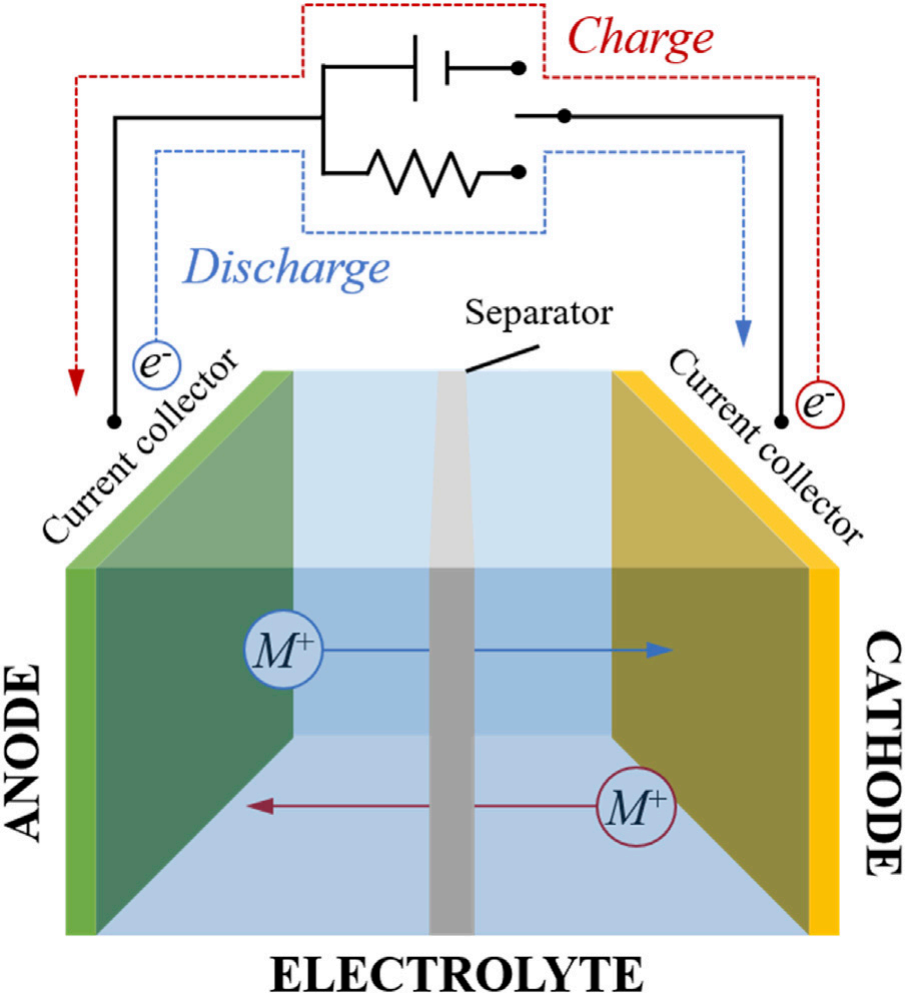
General schematic drawing of a metal (ion) battery operation where the metal can be replaced by lithium or sodium.

A sustainable future implies reinforcing energy savings and using energy sources with low or zero GHG emissions, such as renewables. Although renewable energy sources have important benefits such as being respectful with the environment, safer for our health and unlimited, they also present some drawbacks. An especially critical factor is related to their intermittency, that is, the moments of maximum production do not match with those of higher demand, which requires the need to implement systems that can store the excess energy and deliver it in times of need. Electrochemical energy storage (EES) devices are the most promising solution to address the problem of intermittency and support the implementation of renewable energies. Among all EES systems, lithium-based batteries are the most exploited ones, particularly lithium-ion batteries (LIBs) (Jiang et al., 2021). LIBs provide higher voltage and specific energy than the batteries based on other chemistries, such as zinc or lead, because of the low reduction potential of Li^+^/Li and the relatively light weight of lithium, respectively. Moreover, the small size of lithium cations makes the host cathodes/anodes more stable during charge and discharge cycles, due to a lower distortion in the electrode structure during electron transfer. The charging and discharging mechanism in LIBs relies on the reversible shuttling of lithium ions between the two electrodes, as shown in Figure 1. Lithium cations are de-intercalated from the active material in the cathode when LIBs are charged. Then, the cations move through the electrolyte and are reduced at the anode. When LIBs are discharged, lithium ions are oxidized at the anode, passing through the electrolyte and intercalating in the cathode material. The efficiency of these processes defines the overall efficiency of the battery (Mehek et al., 2021). Regarding the selection of materials, commercial LIBs use as active materials in the cathode mainly layered oxides such as LiCoO_2_ and Li [Ni_1-x-y_Mn_x_Co_y_]O_2_, the LiFePO_4_ polyanionic material, or the LiMn_2_O_4_ spinel (Nayak et al., 2018). Among the materials implemented in the anode region, carbonaceous structures such as graphite stand out, although there are also other promising materials to store lithium such as metal alloys with metals such as Si or Sn or conversion-type anode materials such as transition metal oxides or chalcogenides (Nzereogu et al., 2022). In recent years, the great potential of silicon has been demonstrated by delivering a capacity one order of magnitude higher than the graphite (Palomares, Nieto and Rojo, 2022). Finally, commercial LIBs usually use non-aqueous solutions as electrolyte based on a lithium salt (lithium hexafluorophosphate) dissolved in organic carbonates (generally a mixture of ethyl carbonate and dimethyl carbonate) (Takada, 2018).

Although LIBs have been successfully commercialized for a wide range of applications, lithium is considered a critical element due to its low availability and limited number of reserves. In addition, the exploitation of lithium may not cover the future demand. Given these problems, it is important to search for alternatives to LIBs, such as the use of other metallic elements such as sodium. Sodium and lithium share some chemical characteristics, which together with the abundance of sodium reserves, has driven the development of sodium-ion batteries (NIBs) as a promising alternative to LIBs (Goikolea et al., 2020). Although the chemistries of lithium and sodium are quite similar, the large size of the sodium cation relative to the lithium one makes it necessary to adapt LIB materials to translate them into NIBs. Thus, in the cathode of commercial NIBs, the layered transition oxides such as NaNi_0.3_Fe_0.4_Mn_0.3_O_2_ or Na_a_Ni_(1−x−y−z)_Mn_x_Mg_y_Ti_z_O_2_, the polyanionic Na_3_V_2_(PO_4_)_2_F_3_ or the Prussian blue analogs such as Na_x_MnFe(CN)_6_ stand out, while in the anode, the materials that are being exploited are mainly hard carbons (Abraham, 2020; Goikolea et al., 2020). Today there is already a large-scale production of NIBs and LIBs, but certain characteristics such as autonomy, fast charging and useful cycling still need to be improved. In addition, there are safety concerns due to the use of liquid electrolytes. The most common electrolytes are composed of lithium or sodium salts, such as LiPF_6_ or NaPF_6_, dissolved in organic carbonates (Li et al., 2016). This type of solvents are flammable, which implies an inherent risk of explosion or combustion of the battery. They also exhibit a certain level of toxicity and therefore the device must be sealed tightly to prevent leakage, which is detrimental to the environment. In addition, it should be noted that the use of liquid organic electrolytes reduces the useful life of batteries since their components are degraded during consecutive cycling. As a strategy to avoid these safety problems, the development of batteries based on solid state electrolytes (SSEs) was proposed, exploring mainly three types of solid electrolytes: solid polymer electrolytes (SPEs), inorganic solid electrolytes (ISEs), and composite solid electrolytes (CSEs) (Chen A. et al., 2020; Zhao Q. et al., 2020). SSEs show improved thermal stability and can operate in extreme conditions, in a temperature range of -50–200 C or even higher (Wang L. et al., 2020). Furthermore, they allow to reduce the size of the device, increase the mechanical resistance of the battery as well as the energy and power densities and improve the electrochemical stability (Jetybayeva et al., 2021). Another important advantage of using SSEs is their ability to prevent the growth of dendrites, in addition to improving serious safety issues (He et al., 2019). Although impermeability against lithium/sodium anodes is always mentioned among the benefits of solid electrolytes, this statement is not always true. When implementing an SSE in order to avoid the growth of dendrites (or filaments), there are several factors to take into account, such as the chemo-mechanics of the formation of the anode/electrolyte interface and its evolution throughout the charge/discharge processes, the pressure established in the assembly and operation of the battery or the purity of the metallic anode surface (Hatzell et al., 2020).

Designing an advanced solid-state electrolyte faces a major challenge (Ma and Tietz, 2020; Zhao Q. et al., 2020). In addition to complying with thermal stability in a wide range of operating temperatures (it should not be forgotten that the battery in its natural operation emits heat) (Xu and He, 2014) and a high level of ion transport, it must be mechanically robust enough to prevent the growth of dendrites, but also flexible enough not to crack during handling processes (Takada, 2018). Furthermore, two of the main challenges related to the implementation of SSEs are their low ionic conductivity (Yang and Wu, 2022) and the formation of an inefficient electrode-electrolyte interface (Chen et al., 2020; Gao et al., 2022). The function of the electrolyte is to build a channel that enables the movement of metallic ions between the anode and cathode during charge and discharge cycles. Therefore, if the ionic conductivity of the electrolyte is not sufficient, the movement of the ions will be too slow, giving rise to an inefficient device (Zhao et al., 2019). Among the different materials investigated, those based on inorganic materials seem to have very promising properties, such as ionic conductivities higher than 10^–4^ S cm^−1^ and an extended electrochemical stability window compared to conventional organic electrolytes (Goikolea et al., 2020). The main problem of these materials is their poor electrode/electrolyte interface. The reduced contact between both components produces a high impedance at the interface that translates into a very poor electrochemical performance (Gao et al., 2018).

**FIGURE 2:**
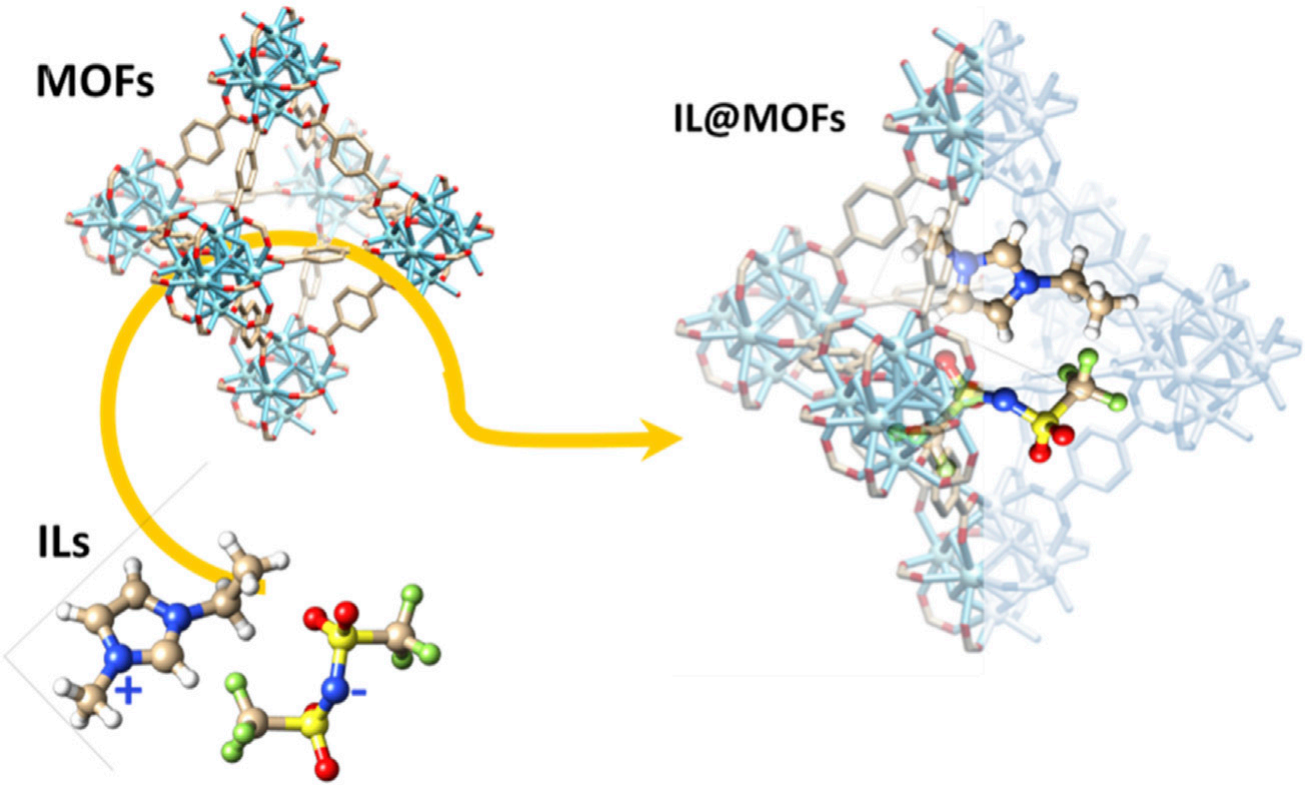
Representation of an illustrative image of the MOFs, ionic liquids and the possible combination of both into IL@MOF type composite materials.

In recent years, the development of energy storage devices based on new families of materials such as metal-organic frameworks (MOFs) has drawn increased attention (Zhao et al., 2020; Cui et al., 2021; Jiang et al., 2021). MOFs have a three-dimensional porous crystalline structure composed by different metal ions or aggregates bonded to organic ligands. These materials have been widely investigated in other applications such as gas adsorption (Shan et al., 2017) and heterogeneous catalysis (Fidalgo-Marijuan et al., 2018) (Figure 2). Benefiting from rich porosity, controllable functionality and modularity, MOFs not only offer great opportunities to manipulate the physicochemical and electrochemical properties of SSEs in LIBs, but also provide ideal structures to investigate ionic conduction mechanisms and structure-property relationships due to their adjustable porous structure and high surface area. One of the greatest challenges in the use of MOFs is the synthesis of this type of materials, and although today the range of reported crystalline structures is very wide and their synthesis conditions are well known, the cost and sustainability (associated with use of toxic products) in the production of this type of compounds continues to be a challenge. For the correct use of these materials in different applications, the control of the crystallinity and the surface area of the materials obtained are critical. Nowadays, in addition to the classic synthesis methods for this type of compounds, such as crystallization by slow evaporation or hydro/solvothermal synthesis, new methods have been developed aimed to reduce synthesis times (microwave or ultrasound synthesis), to reduce or avoid the use of solvents (mechanochemical synthesis) or reduce the temperature (electrochemical synthesis). The use of this new family of versatile materials may facilitate obtaining better ionic conductivities, but they will still suffer from poor interfacial contact. To solve this fact, a smart strategy is the introduction of ionic liquids (ILs) in the porous network that promote good ionic conductivity and allow better contact at the interface (Figure 2). ILs are molten salts composed of charged ions (cations and anions) with a melting temperatures below 100 C (Qi et al., 2020; Liu et al., 2021). Usually, cations are the derivatives of 1-methylimidazole and the anions are the conjugate base of an inorganic acid, such as tetrafluoroborate or hexafluorophosphate. Compared to conventional organic liquids, the intermolecular forces of ILs are driven by the strong ionic bond that makes ILs to exhibit both a high lattice energy and melting temperature. ILs present low flammability, low volatility, thermal stability and an electrochemical stability window up to 6.0 V (Liu et al., 2021; Majid et al., 2022).

**FIGURE 3:**
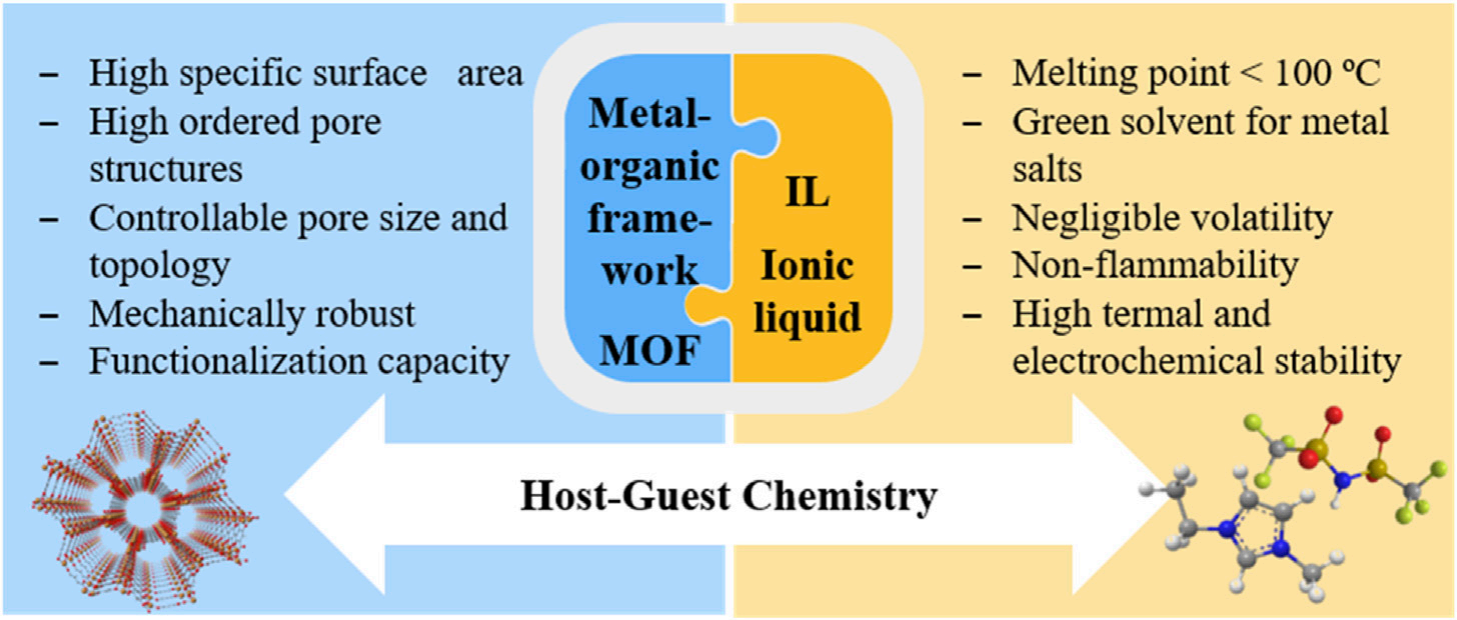
Overview of the fundamental properties that MOFs and ILs present for their integration as components of IL@MOF hybrid electrolytes.

Figure 3 summarizes the main properties of both MOFs and ILs, with a view of combining them to produce (quasi)solid hybrid electrolytes. The inclusion of ILs into a metal-organic framework (IL@MOF) has currently emerged as an interesting class of hybrid material that can offer excellent electrochemical properties (Majid et al., 2022). Owing to their unique porosity properties, MOFs can enable host-guest chemistry via the insertion of guest ions or molecules and improve the overall electrochemical performance. IL@MOFs share the dynamic behavior of liquid electrolytes, due to the presence of the IL, with MOFs as a solid support material. IL@MOFs have shown high ionic conductivity and the potential to be used as SSEs even at relatively low temperatures without diminishing the metal ion diffusion (Majid et al., 2022). This type of hybrid electrolytes based on the combination of a MOF with an IL allow the use of a metallic anode, which would tremendously boost both volumetric and gravimetric energy density values and pave the way towards next-generation lithium (or sodium) metal batteries (Wang R. et al., 2020). With a theoretical specific capacity as high as 3,860 mAh g^−1^ (for metallic Li) or 1,166 mAh g^−1^ (for metallic Na) (Xu et al., 2014; Sun et al., 2020), as well as the lowest known standard reduction potentials ( $E_{Li^{+}/Li}^{0}$ = -3.04 V; $E_{Na^{+}/\mathrm{Na}}^{0}$ = -2.71 V vs. standard hydrogen electrode) alkaline metals are considered indeed the ideal high-capacity anodes.

The use of IL@MOFs-type composites as electrolytes in rechargeable batteries is a very recent topic. The first published studies date back to 2018 (Wang et al., 2018a; Wang et al., 2018b). Although the proof of concept has already been demonstrated, there is still a large gap between laboratory results and the commercialization of batteries based on this type of electrolytes. This review aims to bridge the laboratory-market gap by analyzing the published data on the use of different combinations of MOFs and ILs. Furthermore, the combination of IL@MOF composite materials with different polymeric matrixes will also be explored herein. These composites aim at improving the performance of SPEs and advance on the fabrication of a new generation of all-solid-state batteries.

## Metal-organic frameworks as active materials for metal (ion) batteries

MOFs are porous coordination polymers in which the metal centres (or metal clusters) are linked through organic ligands. By a correct selection of both the metallic ion and the organic connector, it is possible to tune the properties of the material. Thus, it is possible to control the porosity, the size of the pores and their three-dimensional arrangement and topology. In addition, the Lewis acid character of the metal centre makes it possible to control the host-guest chemistry that is induced by introducing different compounds such as ILs into the channels. The great adaptability of these structures together with their high degree of design possibilities allows the implementation of MOFs in practically any type of device including rechargeable or secondary batteries, the focus of this review. Rechargeable batteries are one of the main EES devices and are in the spotlight of many researchers. The electrochemical performance of current batteries can be ameliorated as electrode materials have still much room for improvement. An electrode material must possess structural stability and a low overpotential, exhibit reversible redox reactions and, above all, must have a good ionic/electrical conductivity (Cui et al., 2021). In this sense, MOFs provide metal centres that can hold multiple electrons, and thanks to the organic ligands, nanometer-sized cavities and open channels that facilitate electrolyte access and the diffusion of metallic ions (Zhang et al., 2019). This way, metal ions from the electrolytes can be by reversible inserted/de-inserted into the pores (Jiang et al., 2021), and due to the large void spaces and high structural flexibility, MOFs can also support volume expansions. Consequently, MOFs can also be used as anodes or cathodes, not only delivering high reversible capacities and energy densities but also exhibiting good cyclabilities (Férey et al., 2005; Song and Zhou, 2013; Wu et al., 2015; Zhang et al., 2019; Mehek et al., 2021).

MOFs have been explored for use as electrodes, but there is still much room for improvement because the direct use of MOFs as electrode materials for LIBs has some limitations, such as their low electrical conductivity, poor capacity retention or low cycling performance (Petit and Bandosz, 2009; Liu and Tang, 2013; Zhu, Wang and Zuo, 2019; Jiang et al., 2021).

To overcome these disadvantages, MOF-based composites for electrode materials have been investigated. MOFs can be synergistically combined with a great variety of materials like carbon-based materials, semiconductor silicon or phosphorus, among others (Zhang et al., 2019). The most relevant MOF-based composites are those based on Co, Mn, Fe or Cu (Gou et al., 2014; Jiang et al., 2021). Additionally, MOFs can also be used as starting reagents or templates for the production of transition metal oxides, carbonaceous materials and MOF/C-type materials with potential applications in LIBs (Zhang et al., 2019; Zhu, Wang and Zuo, 2019).

**FIGURE 4:**
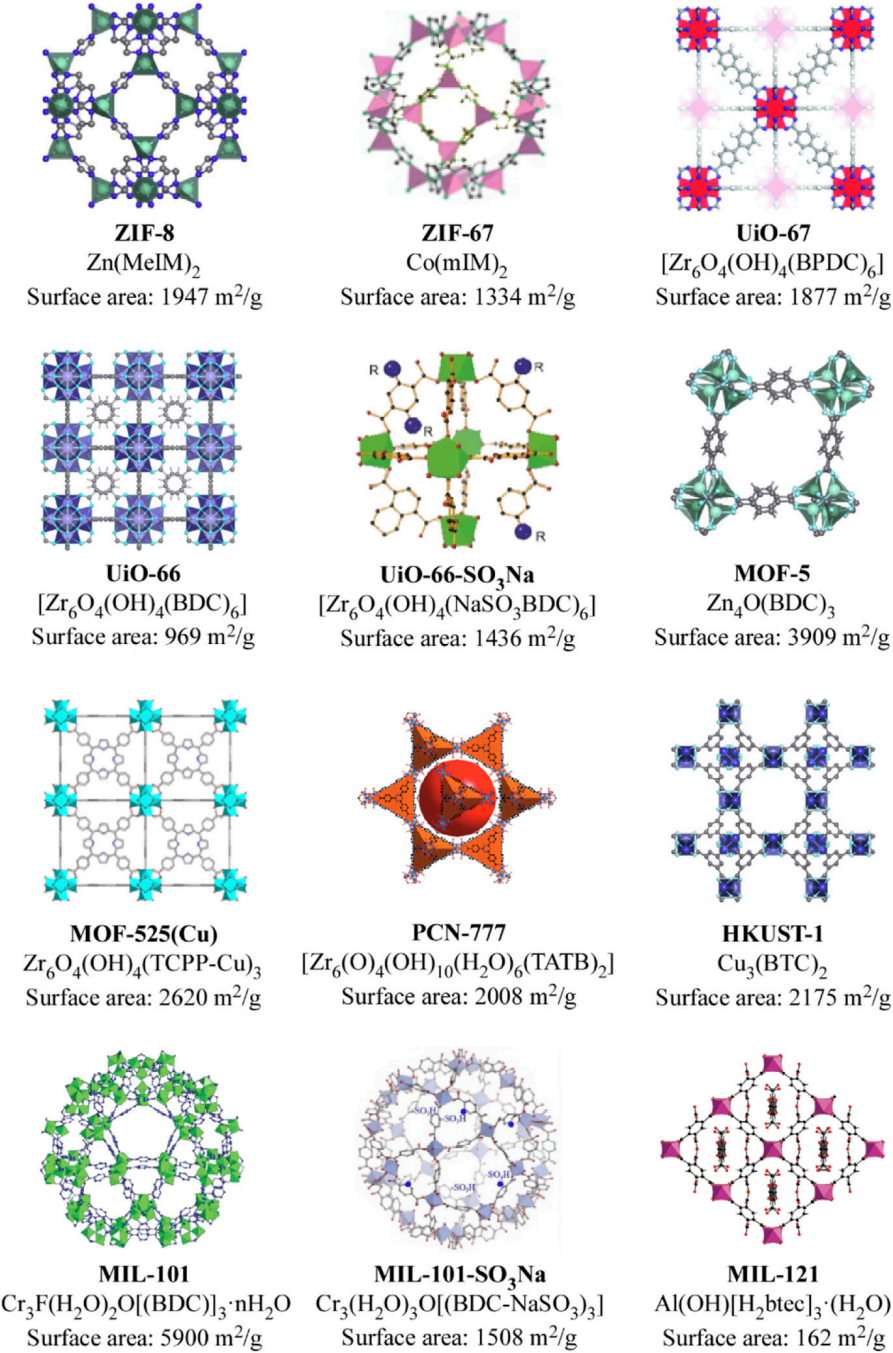
Summary of the characteristics of MOFs used for IL incorporation as electrolytes in sodium and lithium based batteries. (Férey et al., 2005; Banerjee et al., 2008; Volkringer et al., 2010; Chavan et al., 2012; Feng et al., 2015; Shaikh et al., 2019; Oozeerally et al., 2021; Yang and Kou, 2021).

Apart from the use of MOF type materials in the electrodes, MOFs can also be utilized as electrolytes due to their intrinsically versatile properties, and they have proven to be an effective and viable alternative. These three-dimensional networks have a predominantly insulating character and, to be used as electrolytes, it is necessary to ensure the movement of ions between the electrodes. This mobility is possible through the introduction of liquid electrolytes into the pores of the MOFs, among which ILs stand out. ILs arise from the combination of a large asymmetric organic cation (e.g. imidazolium or pyrrolidinium) with an anionic species that can exhibit an inorganic or organic nature (e.g. tetrafluoroborate, hexafluorophosphate, or bromide) (Kinik, Uzun and Keskin, 2017). ILs are considered green solvents characterized by low melting temperature, high solubility for certain salts, negligible volatility, non-flammability and, what is more important, high electrochemical stability (Clark et al., 2018). By introducing ILs into the MOF pores, it is possible to store a high density of charged species in a very small volume (He et al., 2019). Furthermore, these charged species will be very close to each other in the small pores of the MOF, which will facilitate the movement of the cations. In this way, the MOF will act as a separator that electronically isolates the electrodes, supports a large amount of liquid electrolyte and physically prevents the growth of dendrites. The IL, on the other hand, will provide high ionic conductivity in addition to exhibiting low internal resistance. The inhibition of dendrite growth is one of the strong aspects that support the use of MOFs as electrolytes or separating membranes. Dendrites are associated not only with safety hazards (internal short-circuits) but also with high polarization due to the formation of a porous and uneven SEI (solid electrolyte interphase). Furthermore, dendrites involve a loss of active Li metal through reactions with the electrolyte. The IL-laden MOF provides ordered ion transport leading to more stable metal electrodeposition. The properties of the IL@MOF composite are determined by the size, shape and chemical environment of the MOF pores. In this sense, the structures of the most common MOFs together with the dimensions of the pores are displayed in Figure 4.

**FIGURE 5:**
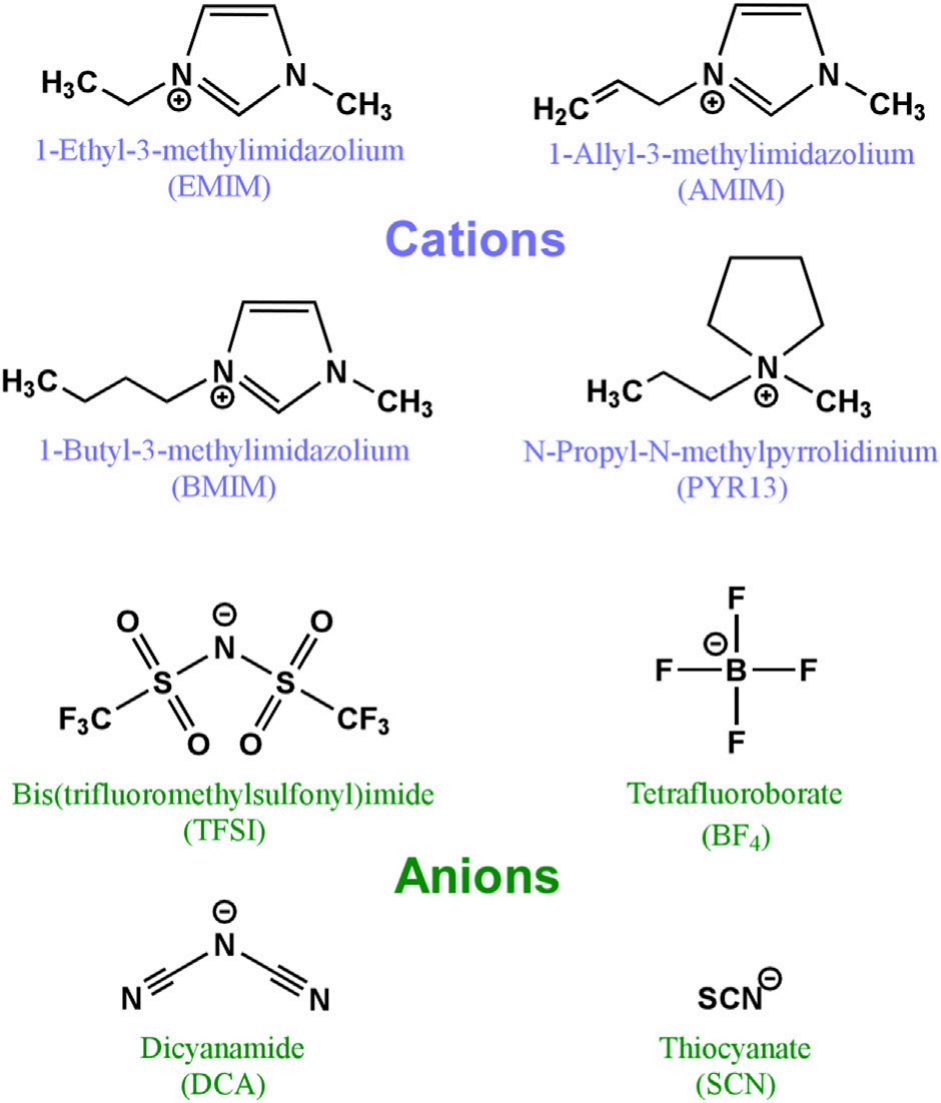
The most common ILs used as MOF pore fillers for electrolytes in sodium and lithium based batteries.

The transport of ions in the hybrid electrolyte can be facilitated by modifying the structures of the MOFs with different functional groups, such as amino groups that are capable of interacting with the ions in the electrolyte (Liu et al., 2017). It is worth noting that MOFs have a metallic centre that acts as a Lewis acid and can bond with the Lewis bases of the IL. Thus, the charged species of the IL can not only be confined to the MOF pores due to their size, but can also be trapped through chemical bonds. This way, by a correct selection of the structure of the MOF and the IL to be introduced into the pores, it is possible to develop single-ion conductors. Regarding the IL, the size of the loaded segments is critical since they must be able to be introduced into the pore where they will be confined. Figure 5 shows the most used ions for their implementation in this type of hybrid electrolytes.

The correct performance of the IL@MOF hybrid electrolyte is directly related to the interactions established between the polar surfaces of the MOF and the ions from the IL. Furthermore, the MOF must be stable towards the IL. Finally, another critical factor, as will be discussed later, is related to the amount of IL that is stored in the MOF cavities. To facilitate the introduction of ILs into the pores, mild heat treatments (80–100°C) are usually applied, which will favor the diffusion of the species. There are different strategies to form these hybrids as presented in different recently published reviews (Kinik, Uzun and Keskin, 2017; Zhao R. et al., 2020). The investigation of these IL@MOF hybrid electrolytes is in an early stage, but there are already works that demonstrate the viability of these materials for their implementation in all-solid-state batteries.

## Implementation of IL@MOF hybrid solid electrolytes in Li and Na-based batteries

The protocol followed by different authors for the implementation of this type of hybrid electrolytes in batteries is quite similar. First, the synthesis of the host MOF to which the IL is inserted into its pores is carried out, grounding together the MOF with the IL and the mixture is heated between 80 and 150°C to favour the capillary action since heating promotes the diffusion of the IL through the MOF pores (Fujie and Kitagawa, 2016). Other strategies applied to obtain IL@MOF focus on soaking a pre-synthesized MOF with the IL or introducing IL precursors into the MOF pores that will be assembled within the pore itself (Zhao R. et al., 2020). Each IL@MOF combination must be carefully analysed, being particularly critical the study of the amount of IL used in the mixture with the MOF. Small amounts of IL will have a low impact on the conductivity of the hybrid system, since MOFs alone tend to act as insulating materials (Zhao R. et al., 2020). On the other hand, an excess of IL gives the IL@MOF mixture a wet gel appearance. In general, the aim is to obtain a “free flowing” dry powder, a sign that the IL is completely encapsulated in the MOF pores. The preparation of IL@MOF mixtures is usually carried out either based on the weight percentage of the components (the most common method) or taking into account the percentage of the free pore volume of the MOF occupied by the IL. In order to establish the correct filling of the pores with the IL, different techniques are usually used, such as infrared spectroscopy. In this way, it will be possible to establish the presence of IL in the mixture, but it is not possible to determine if the IL is inside the pores or coating the surface of the MOF particles. The correct introduction of the IL in the pores can be recorded by means of X-ray diffraction, where a change in the relative intensity of certain characteristic maxima of the MOF will be observed, which is related to a change in the electronic density in the MOF pores due to the introduction of the IL (Fujie et al., 2014; Xu et al., 2019). The decrease in intensity of certain maxima (generally the highest intensity peaks) with the introduction of the IL is associated with an increase in the disorder in the MOF structure or with the presence of mobile ions in the pores (Yoshida et al., 2019). Through the analysis of the N_2_ gas adsorption/desorption isotherms, it is also possible to determine the degree of insertion of the IL in the pores, since the uptake of N_2_ decreases rapidly as the filling level of the porosity increases. After the physical-chemical characterization of the IL@MOF mixtures, the ionic conductivity in symmetric metal|IL@MOF|metal systems is studied using electrochemical impedance spectroscopy (EIS). These measurements can also be achieved by placing a pellet of IL@MOF material between two stainless steel blocking electrodes. In EIS measurements, an amplitude between 5–50 mV is usually applied, required to ensure a linear behavior of the current according to the Butler-Volmer model, and a sweep is performed at frequencies usually between 0.01 Hz and 1 MHz (Bredar et al., 2020). The ionic conductivity values are determined from the analysis of the recorded Nyquist diagrams, measuring the resistance associated with the material under study. If the appearance of a characteristic semicircle is appreciated, the resistance is calculated by means of the difference between the two points of contact of the semicircle with the real impedance axis. Sometimes, when it is not possible to observe that semicircle, the point of intersection of the diffusion tail with the real impedance axis is taken into account. This way, it is possible to calculate the ionic conductivity considering all of the mobile ionic species through the following equation (Nozari et al., 2020):

$$\mathrm{\sigma =}\frac{L}{\mathrm{RxS}}$$ (1)

where, σ refers to the ionic conductivity, R is the resistance related to the hybrid electrolyte IL@MOF, L is the thickness of the electrolyte pellet, and S is the area of the pellet. When these measurements are carried out at different temperatures, it is possible to determine the activation energy of the processes involved by means of the Arrhenius equation (Aziz et al., 2018):

$$\mathrm{\sigma =Α}\exp\left(\frac{-E_{a}}{\mathrm{kT}}\right)$$ (2)

where, Α is a pre-exponential factor, $E_{a}$ refers to the activation energy, k is Boltzmann’s constant and T is the temperature at which the measurement was recorded. This activation energy provides information on the ease with which ion transport will occur and even on the type of mechanism responsible for conduction.

The ionic conductivity will depends on different factors, the pore size of the MOF being one of the most critical ones. The IL must enter the MOF pores, where different IL-MOF interactions will occur depending on the nature of both components: Coulombic-type forces, Van der Waals-type interactions, hydrogen bonding, and coordination interactions, among others (Fujie and Kitagawa, 2016). For this reason, it is critical to adjust the dimensions of the MOF pore to the size of the IL. The polar surfaces of the MOFs will allow a selective bonding with the ILs, which will influence the ionic conductivity and the ion transfer number. Correctly controlling the strength of the MOF-IL interactions can facilitate higer mobility of the charged species through the MOF channels. In fact, the interionic interactions between the anionic segment of the IL and the metallic centre of the MOF are capable of perturbing even the symmetry of the MOF (Dhumal et al., 2016). Inside the channels, the charged segments form different layers, being the outermost (in contact with the walls of the MOF pores) the one that forms a first ion-pair configuration due to the aforementioned interionic interactions. The second layer exhibits much weaker interactions between the IL ions, which allows a higher mobility of the charged species since they are more loosely bound to the MOF structure. Through computational studies based on ab initio calculations, the important role that the nature of the IL anions plays in the charge transfer and stability of the IL@MOF structures has been established (Thomas et al., 2019). Computational modelling has even determined that ionic mobility is strongly affected by the degree of occupancy of the pores. Be aware that the mobile cations and anions in the MOF pores move in opposite directions throughout the battery cycle. If a high loading of IL occurs in the MOF, significant interactions between the ions will occur. In high IL concentrations, cations and anions, traveling in opposite directions when an external electric field is applied, block each other’s pore windows, since the rigid structure of the pores and the size of the pore window prevent the simultaneous passage of ions (Kanj et al., 2019).

It is important to distinguish between the ionic conductivity of the IL@MOF and the mobility of the corresponding metal ions (Li^+^ for example in the case of LIBs). The ionic conductivity takes into account the mobility of both the corresponding metal ions and that of the segments that make up the IL; but, in order to apply the IL@MOF system, it is essential to know how the metal cations diffuse through the electrolyte. This last parameter is related to the transference number, defined as the fraction of the ionic conductivity due to the cation of the metal (Fong et al., 2019). This factor is usually calculated through DC polarization curves in which a constant DC polarization voltage of 10 mV is applied, recording the impedance spectra at the beginning of the measurement and in stable conditions after polarization. Subsequently, the Evans equation (Evans, Vincent and Bruce, 1987) is used:

$$t_{M^{+}}=\frac{i_{\infty }\times \left(\Delta V-i_{0}R_{0}\right)}{i_{0}\times \left(\Delta V-i_{\infty }R_{\infty }\right)}$$ (3)

where, $t_{M^{+}}$ is the transference (or transport) number of the metal cations, ΔV is the applied potential difference (usually 10 mV), $R_{0}$ and $R_{\infty }$ correspond to the resistance values before and after the polarization experiment, respectively, $i_{0}$ is the current recorded in the beginning of the experiment and $i_{\infty }$ refers to the current value obtained in stable conditions after polarization. Other authors follow the method proposed by Hu et al. (Suo et al., 2013) for the calculation of the cation transference number, since this method is less dependent on the formation of the solid interface between the electrolyte and the metallic electrodes (Oteo et al., 2019). The transference number is defined as the fraction of current carried by a given species (the metal cation in this case) in a system without concentration gradients. The closer the value is to 1, the greater the diffusion of the cation. In general, low $t_{M^{+}}$ values refer to systems in which the counterion is highly mobile and the cation, on the other hand, has a much more limited mobility due to the solvation shell that surrounds it. The IL@MOF hybrid electrolytes, if carefully designed, can help improve the transference number. On the one hand, the angstrom-scale pores make it possible to restrict the mobility of the solvent ions, that is, the ions of the IL, which will remain confined in the cavities in the structure of the MOF. On the other hand, the metallic centers of the MOFs act as Lewis acids capable of “trapping” the anions of the medium (Lewis bases), thus improving the mobility of the metallic cations. Using nuclear magnetic resonance (NMR) it is also possible to establish the self-diffusion of the cations under study (Zettl and Hanzu, 2021). The line width depends on the mobility of the metal cations. In this sense, a narrower line width leads to faster ion exchange processes. This phenomenon is called motional line narrowing.

One of the main objectives of the use of solid electrolytes is related to the inhibition of the growth of dendrites in the cycling of the batteries. In order to verify whether hybrid electrolytes meet this requirement, measurements are carried out monitoring the metal plating/stripping processes. In these experiments, a current density between 0.5 and 0.1 mA cm^−2^ is applied to a symmetrical cell with active metal electrodes, metal|IL@MOF|metal. In this way, it is possible to analyze the stability of the hybrid electrolyte over time against the appearance of short circuits due to the growth of dendrites. In these tests, optimal behavior implies that there are no sudden increases in polarization and the surface of the metallic electrode should be smooth and homogeneous after the test. Another important aspect to consider is the effect of the electrolyte on the operating potential window of the battery, which is analyzed by linear sweep voltammetry. This allows to establish the maximum voltage at which the battery can be cycled. In the case of IL@MOF electrolytes, this potential window is usually delimited by the stability of the IL. Finally, some of the reported works evaluate the performance of the hybrid electrolyte implemented in a solid-state battery. For system assembly, it is common to include portions of IL@MOF in the cathode formulation and to use the fresh metal as the anode. To improve the electrode/electrolyte interfaces it is usual to add a few drops ( ∼ 3 μ L) of liquid electrolyte on both surfaces of the electrolyte.

**FIGURE 6:**
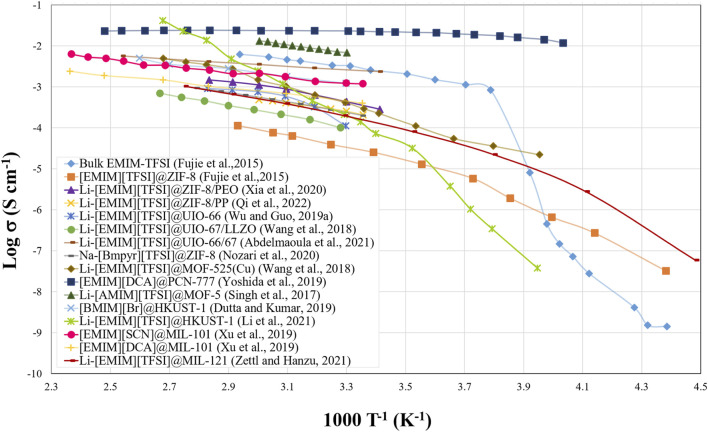
Plot of ionic conductivities versus temperature for different IL@MOF hybrid electrolytes.

Fujie et al. reported for the first time in 2014 a strategy capable of stabilizing an IL within the pores of a MOF (Fujie et al., 2014). In their study, they selected a Zn-based MOF, ZIF-8, to introduce into its pores the 1-ethyl-3-methylimidazolium bis(trifluoromethylsulfonyl)imide IL [EMIM][TFSI]. ZIF-8 features large cavities (1.16 nm) that are interconnected through small openings (0.34 nm). After introducing the IL into the pores, a clear change in the fusion and freezing processes of the IL were observed. Specifically, it was observed that by introducing the IL in the MOF it was possible to stabilize the liquid phase of the IL at lower temperatures. One year later, in 2015, the same group analysed the ionic conductivity of the [EMIM][TFSI]@ZIF-8 system (Fujie et al., 2015b). The IL remains in liquid state inside the MOF cavity even at low temperatures, and it maintains the ionic conductivity even at temperatures that otherwise it would be frozen, as can be seen in Figure 6. This group was also a pioneer in the study of the dynamics of Li^+^ ions, which is directly related to the interactions that may occur between the MOF (the host) and the IL (the guest) (Fujie et al., 2015a). As mentioned above, the analysis of the self-diffusion of lithium cations is important for the integration of the hybrid electrolyte in a LIB, since the ionic conductivity is related not only to Li^+^ but also includes the mobility of the counterions. Lithium was introduced into the hybrid system by mixing the IL with a lithium salt (LiTFSI) obtaining a stoichiometry of [Li_0.2_EMIM_0.8_][TFSI]. The effect of the MOF on the mobility of [TFSI]^−^ anions is related to the glass transitions that the IL undergoes. When the IL is confined in the MOF, these transitions are less abrupt, and at low temperature the mobility is higher than that of bulk [EMIM_0.8_Li_0.2_][TFSI]. This effect on the mobility of lithium cations is not so clear. When lithium is introduced, a slight decrease in the ionic conductivity of the hybrid system (IL@MOF) is observed, accompanied by an increase in the activation energy, which was later related to the increase in the viscosity of the IL when adding the lithium salt (Wu et al., 2013). Regarding the diffusion mechanism of Li^+^, inside the MOF cavities [TFSI]^−^ anions are trapped, since the openings that connect the pores are too small to let the anions pass (0.34 vs. 0.76 nm of the [TFSI]^−^), while lithium ions with an ionic radius of 0.076 nm can move free from one cavity to another. Thus, the anions cannot accompany Li^+^ ions in their diffusion process. Li^+^ cations exchange solvation anions as they move through the MOF pores (Borodin, Smith and Henderson, 2006), following a Grotthuss mechanism similar to that described for protonic conductivity (Cukierman, 2006).

Another UiO-66 and UiO-67 MOFs that have also been explored for the design of IL@MOF-type hybrid electrolytes. Wu and Guo observed, when mixing monodisperse nanocrystals of UiO-66 impregnated with Li [EMIM][TFSI] and polyethylene oxide (PEO), a clear improvement in lithium transference number from 0.18 in the PEO to 0.35 when 20% IL@MOF was added (Zhang Z. et al., 2020). This important improvement in lithium transport seems to be due to the suppression of the crystallinity of PEO when introducing the filler in the composites. On the other hand, the hybrid system Li [EMIM][TFSI]@UiO-67 has been mixed with a solid-state electrolyte based on LLZO (Li_7_La_3_Zr_2_O_12_) in a weight ratio of 20:80 to facilitate the interfacial transport of lithium ions (Wang et al., 2018b). LLZO gaps are filled with the hybrid material, thus, reducing the interfacial resistance, which is verified through the increase in the ionic conductivity.

Core-shell type materials, such as the UiO-66@UiO-67 system, can also be design (Abdelmaoula et al., 2021). This bifunctional MOF-in-MOF system combines a core of UiO-66 with a small pore size where larger ions will be confined (releasing lithium ions to a greater degree) with a coating of UiO-67 with a larger surface area that will stimulate the absorption of the IL. The correct combination of both MOFs together with the optimal amount of IL gives rise to an improvement of the ionic conductivity by almost one order of magnitude with a very small associated activation energy value of 0.086 eV. This fact seems to be related to a better connection of lithium-ion transport channels, giving rise to a transference number as high as 0.63. This hybrid electrolyte has also shown to be stable against the formation of dendrites and can be used in a wide range of current densities (100–1,000 μ A cm^−2^). Furthermore, its performance has been evaluated in a solid state battery prototype with a LiFePO_4_:IL@UiO-66@UiO-67:acetylene black (6:6:2) composite cathode and metallic lithium as anode. In addition to providing high capacity (see Table 1), this battery exhibits a good capacity retention after 100 cycles. Moreover, the battery is able to work at 3C for more than 500 cycles. UiO-66 has also been implemented in Li-S batteries, but in this case the MOF structure has been modified by grafting a sulfonate group (−SO_3_Li) onto the UiO ligand (Chiochan et al., 2020). Thus, the MOF will act as an insulating spacer and provide a pathway for lithium ion transport through the pores. In addition, it is an effective strategy to overcome one of the most critical problems in Li-S batteries related to the migration of polysulfides, since the MOF is capable of acting as a barrier to these species. The presence of the anionic −SO_3_^−^ group induces a repulsion effect on the $S_{n}^{-}$ species and facilitates a jump mechanism of Li^+^ ions, thus, increasing the ionic conductivity. In fact, the higher viscosity of the IL compared to that of usual solvents such as DME (1,2-dimethoxyethane) or DOL (1,3-dioxolane) favours the retention of the polysulfides, preventing their diffusion between the electrodes. This fact is coupled with the pore size of the MOF, which helps suppressing the penetration of these poisonous species. UiO-66 modified with sodium sulfonate groups (−SO_3_Na) has also been evaluated in sodium ion batteries (NIBs) using the 1-butyl-1-methylpyrrolidin bis(trifluoromethylsulfonyl)imide ([Bmpyr][TFSI]) IL enriched with a sodium salt (NaTFSI) (Yu et al., 2021). Through the correct modification of the structure of the MOF ligands, it is possible to modulate the properties of the hybrid system. In this specific case, by introducing the sulfonate group the ionic conductivity at room temperature increases from 0.8 × 10^–4^ S cm^−1^ to 3.6 × 10^–4^ S cm^−1^ because of the easier transport mechanism of Na^+^ ions between these sulfonate groups. On the other hand, although the introduction of sulfonate groups limits the potential window from 5.2 to 4.6 V (vs. Na^+^/Na), the use of high-potential cathodes is still allowed. In fact, in this work the Na|Na-IL/UiOSNa|Na_3_Ni_1.5_TeO_6_ cell is analyzed in a quasi-solid state, showing a remarkable performance with a capacity retention of 76.5% after 100 cycles and with Coulombic efficiencies between 99.0–99.9% (Yu et al., 2021). The IL-charged modified MOF not only has the ability to suppress sodium dendrites, allowing the use of a sodium metal anode, but also aids in the formation of a favourable ionic interface for ion-transfer between the electrode and the electrolyte. In NIBs, the use of other MOFs such as ZIF-8 combined with a sodium-enriched IL [Na_0.1_EMIM_0.9_][TFSI] has also been explored (Nozari et al., 2020). In this work, the effect of the crystallinity of the MOF on the electrolyte conductivity was analysed. Both the crystalline and the partially amorphous (through grinding) MOF present activation energies in the order of 0.26 eV, which suggests that Na^+^ ions are conducted through the MOF micropores following a Gotthuss mechanism and exchanging the solvation TFSI^−^ anions. When partially losing the crystallinity, a decrease in ionic conductivity of one order of magnitude was recorded, but the stability of the electrolyte when exposed to air was higher in the amorphous system, with a lower loss of ionic conductivity.

**TABLE 1:** Summary for IL@MOF based electrolytes.

MOF	Ionic liquid	σ (S cm^−1^)/T (°C)	$E_{a}$ (eV)	$t_{Li^{+}}$	ESW (V)	Capacity (mA h g^−1^)	References
ZIF-8	[EMIM][TFSI]	2.6 × 10^–5^/22	0.35	-	-	-	Fujie et al. (2015b)
	[Li_0.2_EMIM_0.8_][TFSI]	4.4 × 10^–6^/22	0.59	-	-	-	Fujie et al. (2015a)
	Li [EMIM][TFSI]	4.26 × 10^–4^/30	-	0.67	5.2	160 (0.1C)—LiFePO_4_	Xia et al. (2020)
	Li [EMIM][TFSI]	2.09 × 10^–4^/25	0.21	0.45	4.7	152 (C/5) –LiFePO_4_	Qi et al. (2022)
	[Na_0.1_EMIM_0.9_][TFSI]	2.0 × 10^–4^/RT crystalline	0.26	-	-	-	Nozari et al. (2020)
	[Na_0.1_EMIM_0.9_][TFSI]	2.97 × 10^–5^/RT amorphous	0.28	-	-	-	
ZIF-67	Li [Py13][TFSI]	9.9 × 10^–4^/30	-	-	-	151 (0.1C at 60°C)—LiFePO_4_	Chen et al. (2019)
UiO-66	Li [EMIM][TFSI]	3.2 × 10^–4^/25	-	-	-	130 (0.2C)—LiFePO_4_	Wu and Guo, (2019b)
	Li [EMIM][TFSI]	1.3 × 10^–4^/30	-	0.35	-	151 (0.5C)—LiFePO_4_	Wu and Guo, (2019a)
UiO-67	Li [EMIM][TFSI]	4.3 × 10^–4^/RT	0.3	0.45	4.8	149 (0.1C)—LiFePO_4_	Liu and Sun, (2020)
UiO-66@UiO-67	Li [EMIM][TFSI]	2.1 × 10^–3^/RT	0.086	0.63	5.2	163.8 (0.2C)—LiFePO_4_	Abdelmaoula et al. (2021)
UiO-66-SO_3_Na	Na [Bmpyr][TFSI]	3.6 × 10^–4^/RT	-	0.34	4.6	97.5 (C/20)—O′3-Na_3_Ni_1.5_TeO_6_	Yu et al. (2021)
MOF-525(Cu)	Li [EMIM][TFSI]	3 × 10^–4^/25	-	0.36	4.1	145 (0.1C)—LiFePO_4_	Wang et al. (2018a)
PCN777	[EMIM][DCA]	4.4 × 10^–3^/RT	0.20	-	-	-	Yoshida et al. (2019)
MOF-5	[AMIM][TFSI]/LiTFSI	7 × 10^–3^/30	-	-	5.22	3,000—Si anode	Singh, Vedarajan and Matsumi, (2017)
HKUST-1	[Li_0.2_EMIM_0.8_][TFSI]	1.2 × 10^–4^/30	0.56	0.13	-	136.2 (1C)—LiFePO_4_	Wang Z. et al. (2020)
	[BMIM][Br]	1.3 × 10^–3^/RT	0.24	-	6.1	-	Dutta and Kumar, (2019)
	Li [EMIM][TFSI]	0.68 × 10^–4^/25	0.34	0.46	-	144 (0.5C at 100°C)—LiFePO_4_	Li et al. (2021)
MIL-101	[EMIM][SCN]	1.15 × 10^–3^/25	0.17	-	-	-	Xu et al. (2019)
	[EMIM][DCA]	4.14 × 10^–4^/25	0.18	-	-	-	
MIL-101-SO_3_Na	Na [EMIM][BF_4_]	1.32 × 10^–2^/150	0.2	-	-	-	Xu et al. (2020)
MIL-121	Li [EMIM][TFSI]	2.0 × 10^–4^/RT	0.4	-	-		Zettl and Hanzu, (2021)
NbO-Cu-MOF	[BMIM][Cl]	6.63 × 10^–5^/150	1.16	-	-	-	Guo, Song and Wang, (2019)
	[EMIM][Br]	7.5 × 10^–6^/150	1.14	-	-	-	

σ , conductivity; T, temperature; $E_{a}$ , activation energy; $t_{Li^{+}}$ , transference number; ESW, electrochemical stability window; RT, room temperature.

One of the first studies that demonstrated the application of an IL@MOF-type hybrid electrolyte in a rechargeable battery focused on the use of the Zr-based MOF, MOF-525(Cu), impregnated with an IL formulation enriched with a lithium salt [Li_0.2_EMIM_0.8_][TFSI] (Wang et al., 2018a). Once again, the open nature of the MOF allows the introduction of the IL into the cavities, subsequently imposing a confinement of the ionic segments of the IL inside the pores. This confinement decreases the mobility of these species, thus favouring an increase in the transference number of Li^+^ ions, which will be able to move more freely through the channels. In the Li-IL@MOF (where Li-IL stands for Li containing ionic liquid), direct interfacial connection of the internal Li-IL is allowed through the highly porous open network. The interfaces are partially wetted by Li-IL at the atomic level, favouring the transport of Li^+^ ions, which implies a lower resistance to charge transfer. Zr-based PCN-777 MOF has been impregnated with [EMIM][N(CN)_2_] (Yoshida et al., 2019). In this case, the anionic segment of the IL is replaced by dicyanamide, which shows a higher Gutmann donor number than the more usual [TFSI]^−^ anion (41.8 vs. 15.7), which implies that the dicyanamide will have a higher Lewis basicity. Consequently, the anionic segments will be more strongly retained by the metallic centres of the MOF, thus, increasing the ionic conductivity up to a value of 4.4 × 10^–3^ S cm^−1^ at room temperature. According to the study by Yoshida et al. (Yoshida et al., 2019) the first shell of anions will form rigid ionic clusters in the MOF cavities, leaving enough space inside the pores to allow the mobility of the Li^+^ cations.

The IL@MOF hybrid system based on MOF-5 impregnated with the 1-allyl-3-methylimidazolium bis(trifluoromethylsulfonyl)imide [AMIM][TFSI], IL doped with a lithium salt (LiTFSI) was tested with a silicon anode using metallic lithium as reference (Singh, Vedarajan and Matsumi, 2017). In this case, the Li-IL@MOF-5 hybrid system showed a gel appearance, indicating that the amount of IL was higher than that required to fill the pores of the MOF. This excess of IL can be useful in order to obtain higher quality interfaces, that is, a better contact between the electrodes and the electrolyte. In the first cycle, a high irreversible capacity is observed, which is related to the formation of the solid electrolyte interface (SEI), but from the second cycle on, the capacity is practically constant, providing a reversible discharge capacity of 3,000 mAh g^−1^ at 0.1C. Cu (HKUST-1) (Dutta and Kumar, 2019; Li et al., 2021), Cr (MIL-101) (Xu et al., 2019) and Al (MIL-121) based MOFs (Zettl and Hanzu, 2021) have also been studied as ionic conductors.

In general, the studies published to date demonstrate the large potential of solid-state IL@MOF hybrid electrolytes in batteries using metallic lithium or sodium as anodes. Their efficiency in preventing the growth of dendrites has been confirmed. They are also capable of reducing the safety issues associated with the high reactivity of metallic lithium and sodium. MOFs can be custom designed by controlling, on the one hand, the size of the pores, cavities and passage regions and, on the other hand, adapting the host-guest interactions between the MOF and the ions in the channels through the correct choice of ligands and metal centres. In addition, ILs are also chemically tunable by the choice of the constituent cations and anions. With an optimized design, it is possible to obtain electrolytes that not only are a physical barrier to the growth of dendrites but also present an ideal pathway for the transport of metal cations, which will lead to a higher ionic conductivity. The presence of ILs also favours the formation of more ionic and stable SEIs. Another advantage of these materials is their wide electrochemical stability window, which extends up to 5.2 V (depending on the chosen IL, since it is the first component to suffer degradation). This allows the design of solid-state batteries that operate with high-potential cathodes, and thus, can potentially provide high energy density values. Compared to other types of solid electrolytes, the combination of an IL with a MOF has important advantages (Han et al., 2020; Cavers et al., 2022; Guo et al., 2022). The ionic conductivity of the IL@MOF hybrids is higher than that exhibited by the SPEs. In fact, the polymeric nature of SPEs reduces their thermal stability and makes them less efficient in preventing dendrite growth, although they also have simple processability. ISEs, on the other hand, provide a better barrier for dendrites, making this type of inorganic electrolytes one of the safest alternatives. ISEs, as well, are (electro)chemically and thermally stable and have sufficient ionic conductivity (10^–2^ − 10^–4^ S cm^−1^), but give rise to poor electrode/electrolyte interfaces and are difficult to process. IL@MOFs achieve better wetting/contacting at the interface than ISEs and higher impermeability to dendrite growth than SPEs, although their processability, due to the infancy of research on this type of hybrid materials for battery applications, is a critical point, which still needs to be improved. A critical parameter that to date does not seem to have been analyzed in depth is the effect of the electrolyte thickness on the battery performance. Very few studies provide this information, which is usually around 300 μ m (Wang et al., 2018a) and it is important to bear in mind that the mechanical properties of these systems will be closely related to the composition of the hybrid system as well as to its manufacturing process.

## Design and advanced fabrication of IL@MOF solid electrolytes embedded on polymeric matrices

Solid electrolytes based on the use of IL-laden MOFs have proven to be an environmentally benign alternative for the development of solid-state batteries. Although the studies on these IL@MOF composites are highly promising, their implementation as hybrid electrolytes in batteries still faces a huge challenge mainly due to their poor mechanical properties. The rigidity of the composite makes it difficult to handle, being easy to fracture when applying pressure during the cell assembly. These cracks are lethal for the correct performance of the battery. To achieve improved flexibility, some authors have opted for the preparation of membranes in which the IL@MOF composite is incorporated into a polymer, giving rise to a solid polymer electrolyte (SPE) (Guo, Song and Wang, 2019; Barbosa et al., 2021). This approach makes it possible to overcome the different issues faced by current solid polymer electrolytes, such as low ionic conductivity, or defective electrode-electrolyte interfaces. Considering the interesting properties of IL@MOFs, the current state-of-the-art in solid polymer electrolytes technology based on IL@MOF is presented. In this case, the challenge is to produce SPEs with a polymer matrix where the solvent used for the polymer does not affect the physical-chemical and structural properties of ILs@MOFs. In fact, there are just a few works reporting on IL@MOFs dispersed in a polymer matrix. Table 2 summarizes the main advances in IL@MOF-based SPEs according to the polymer and MOF type.

**TABLE 2:** State of the art of the use of IL@MOF for the development of solid polymer electrolytes in lithium-based batteries.

Polymer	MOF	IL	Technique	Main goal/achievement	References
PEO	Cu-based MOF	[BMIM][Br]	Solvent casting	High ionic conductivity value	Dutta and Kumar, (2016)
	UiO-66	[EMIM][TFSI]	Solvent casting	Stable Li plating/stripping	Wu and Guo, (2019a)
	ZIF-8	[EMIM][TFSI]	Solvent casting	Inhibits lithium dendrite growth and enhances Li diffusivity	Hu et al. (2022)
	ZIF-8	(EMIM_0.83_Li_0.17_)TFSI	Solvent casting	Fast ion transport and high ionic conductivity	Xia et al. (2020)
	ZIF-90	Imidazole ionic liquid containing trimethoxysilane groups	Solvent casting	Inhibits lithium dendrite growth	Lei et al. (2021)
PP	ZIF-8	Li [EMIM][TFSI]	*In situ* growth	Inhibits lithium dendrite growth, increases mechanical stability and excellent Li plating/stripping	Qi et al. (2022)
PTFE	MOF-525(Cu)	[EMIM][TFSI]	Solvent casting	Good compatibility against both Li metal and active electrodes; low interfacial resistances	Wang et al. (2018a)
PVDF-HFP	CuBTC	[BMIM][BF_4_]	Solvent casting	Demonstration of the interaction of IL ions with CuBTC-MOF.	Dutta and Kumar, (2020)
	UiO-66-SO_3_H	[EMIM][TFSI]	Solvent casting	Low interfacial resistance and stable Li plating/stripping	Zhang Q. et al. (2020)
	UiO-67	[EMIM][TFSI]	Solvent casting	Low interface impedance	Liu and Sun, (2020)

**FIGURE 7:**
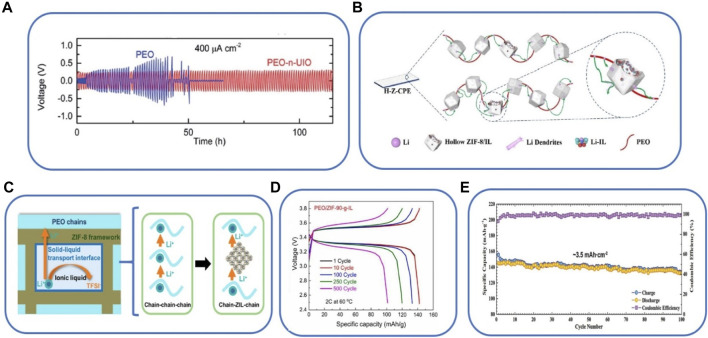
**(A)** Voltage profiles as a function of time for a Li cell at 60°C (Wu and Guo, 2019a), **(B)** schematic illustration of a SPE with PEO and ZIF-8/IL (Hu et al., 2022), **(C)** schematic illustration of the solid−liquid transport interface (Xia et al., 2020), **(D)** charge/discharge curves of lithium batteries using PEO/ZIF-90-g-IL at 60°C and 2C (Lei et al., 2021) and **(E)** cycling performance and Coulombic efficiency of the SPE based IL@MOF at 0.1 C at room temperature (Wang et al., 2018a).

A SPE based on PEO with UiO/Li-IL absorbed onto a UiO-66 MOF shows a stable Li plating/stripping voltage in at 60°C as shown in Figure 7A (Wu and Guo, 2019a). Another SPE composed of hollow ZIF-8 and Li-ILs (ZIF-8/IL) nano-fillers and a PEO matrix has also been developed with high ionic conductivity and improved thermal and electrochemical stability. The schematic representation of this SPE is illustrated in Figure 7B, where the MOFs accommodate Li-containing ILs and maintain stable long-term cycling (Hu et al., 2022). The combination of ZIF-8 with [EMIM][TFSI] and LiTFSI as lithium salt was used to manufacture PEO composites for the development of a new family of solid polymer electrolytes (Xia et al., 2020). PEO, due to its flexibility and low weight, can improve the mechanical properties of IL@MOF hybrid systems. PEO exhibits a very limited ionic conductivity that can be improved as its crystallinity decreases. In the composite, the inclusion of IL@MOF particles help to limit the number of crystalline regions in the polymer. In addition, by studying the transference number in the composite, it is possible to determine that the use of ZIF-8 allows the immobilization of the anionic segments of the IL through Lewis acid-base interactions, releasing lithium cations that participate in the ionic transport. The schematic representation of the nanowetted interface with efficient lithium-ion pathways is shown in Figure 7C. In this work, the Li [EMIM][TFSI]@ZIF-8 hybrid system is integrated in a LIB using LiFePO_4_ as the cathodic active material and obtaining a capacity of 167 mAh g^−1^ at 0.05C. ZIF-8 impregnated with Li [EMIM][TFSI] was also used in the manufacture of membranes, for which ZIF-8 nanoparticles were deposited on the surface and inside of a polypropylene (PP) membrane, which was subsequently soaked with Li [EMIM][TFSI] (Qi et al., 2022). The presence of ZIF-8 increases the stability of the membrane against dendrite growth, which is extracted from the excellent performance in lithium plating/stripping assays. In addition, ZIF-8 seems to be able to provide more effective pathways for Li^+^ transport, giving rise to a decrease in the activation energy down to 0.21 eV. When the membrane is integrated into a LIB, the device shows excellent performance, with a decrease in polarization by the inclusion of ZIF-8 in the membrane. In fact, the presence of the MOF seems to guarantee the uniform deposition of lithium, stabilizing the electrolyte/anode interface, which translates into an improvement in the stability of the battery during cycling.

A new SPE based on zeolitic imidazolate framework-90 grafted with IL (ZIF-90-g-IL) and PEO polymer showed high ionic conductivity and inhibited the growth of lithium dendrites. The charge-discharge curves measured at 60°C and 2C are shown in Figure 7D (Lei et al., 2021). Another novel SPE based on IL-impregnated MOF nanocrystals (Li-IL@MOF) and polytetrafluoroethylene (PTFE) polymer exhibited a high ionic conductivity of 0.3 mS cm^−1^ at room temperature (Wang et al., 2018a). The corresponding battery performance is shown in Figure 7E. An SPE based on poly (vinylidene fluoride-co-hexafluoropropylene), PVDF-HFP, polymer, CuBTC MOF and [BMIM][BF_4_] IL. PVDF-HFP was dissolved in acetonitrile, stirring for 20 min at 50°C. Then, IL@CuBTC nanoparticles were dispersed in the PVDF-HFP solution under ultrasonic treatment for 2 h. Finally, the solution was casted in a glass plate and dried at room temperature (Dutta and Kumar, 2020). The interaction of the ions from the IL and CuBTC-MOF was explored by Fourier transform infrared spectroscopy (FTIR) and X-ray photoelectron spectroscopy (XPS), demonstrating the movement of BF_4_^−^ anions from the IL towards the Cu metallic nodes from the MOF. This SPE shows an ionic conductivity of 8.7 mS cm^−1^ at ∼107°C when 50 wt% of IL is incorporated (Dutta and Kumar, 2020). Another SPE based on PVDF-HFP was produced by adding UiO-66-SO_3_H and [EMIM][TFSI], showing a high ionic conductivity of 1.06 mS cm^−1^ at 25°C, a wide electrochemical stability window from 2.0 to 4.5 V and stable charge/discharge capacities from −20 to 60°C (Zhang Q. et al., 2020). For the same MOF, another SPE based on PVDF-HFP has also been developed (Liu and Sun, 2020). In this work, PVDF-HFP was dissolved in acetone and the IL@UiO-67 filler was dispersed in DMAc. Then, both solutions were mixed and stirred for 2 days and casted on a glass plate. The solvent was slowly evaporated at room temperature, and then using a vacuum oven at 80°C overnight. This SPE showed that MOF nanopores limit the movement of long-chain ions, whereas in the polymer matrix ionic channels are formed allowing the movement of lithium ions. This SPE shows an ionic conductivity of 0.43 mS cm^−1^ at room temperature and delivers a discharge capacity of 149 mAh g^−1^ within 300 cycles at 0.1 C (Liu and Sun, 2020).

When trying to combine an IL with a polymer, the two materials are not always compatible. By introducing the IL into the pores of the MOF, the compatibility between the polymer and the IL improves, since the MOF confines the different ionic parts of the IL and, then, disperses them homogeneously in the polymeric matrix. The MOF provides a continuous path that favours the transport of lithium ions while the polymer matrix, with its flexible nature, provides better mechanical properties by preventing the appearance of cracks in the electrolyte. Ultimately, this allows the battery to present an improved cyclability and a satisfactory capacity retention.

## Conclusion and perspectives

In this review, the main advances in the development of hybrid electrolytes produced from the combination of MOF with ILs have been highlighted. Solid electrolytes are a critical component in next generation rechargeable electrochemical energy storage devices. This solid-state approach avoids the use of flammable solvents, reducing the risks associated with the organic solvents used in conventional electrolytes. Furthermore, the robustness of the MOFs inhibits the dendrite growth, while the ionic liquid provides enhanced ion mobility. Metal cations (Li^+^ or Na^+^) move through the periodically well-organized channels of the MOF, which facilitates a more homogeneous and orderly deposition/release of the metal in the charge/discharge processes. These solid electrolytes allow using a metallic anode, which is another key advantage in the search of high energy density batteries. Moreover, these IL@MOF electrolytes have extended electrochemical stability windows even above 5 V (vs. Li^+^/Li), which enables the use of high-potential cathodes. All these advantages have attracted considerable attention to this emerging research topic.

**FIGURE 8:**
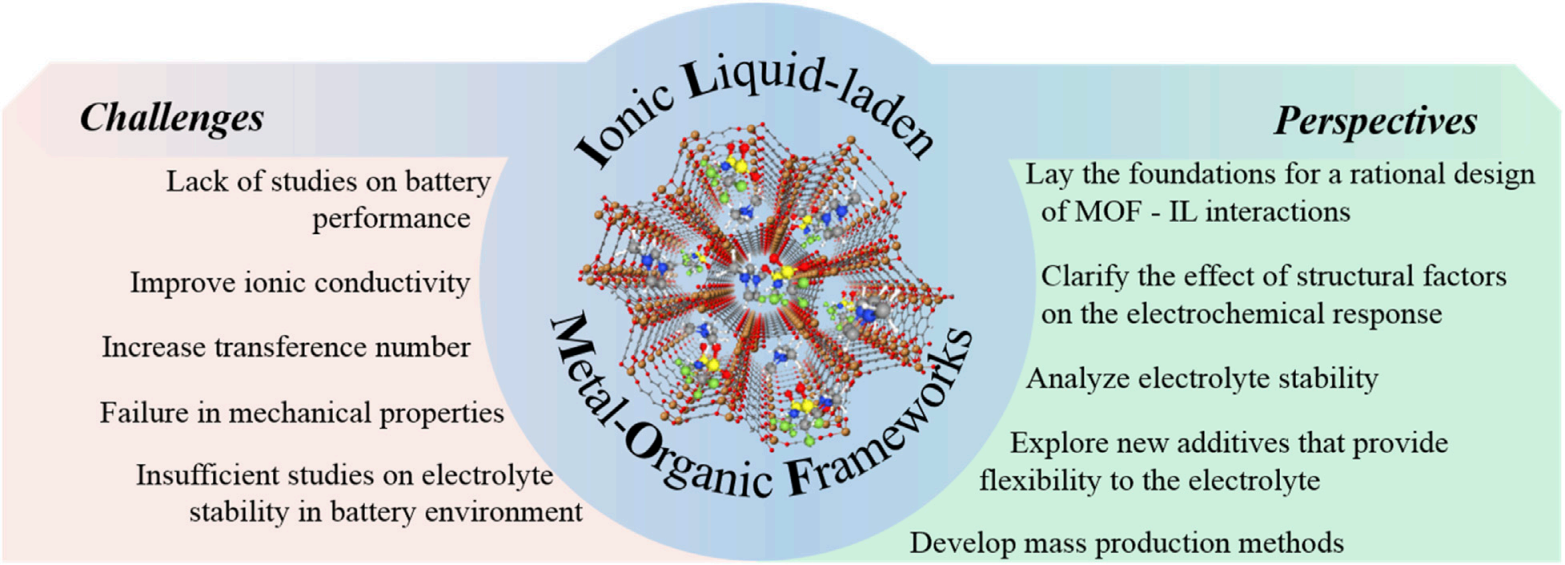
Challenges and perspectives for the development of IL-laden MOFs as (quasi)solid-state electrolytes.

Since the first study about an IL stabilized inside the pores of a MOF, back in 2014, several authors have explored the potential of IL@MOFs as hybrid electrolytes for batteries. The MOF acts a physical barrier to dendrite growth and it is also capable of generating interactions with the counterions from the IL. These interactions cause the ionic segments of the IL to be restricted within the pores, facilitating the movement of lithium or sodium cations. The mobility restriction causes only Li^+^ or Na^+^ to move through the pores, which results in an increased transference number. Following this strategy, it is possible to play with the host-guest chemistry by modifying the Lewis acidity of the metal center, the ligands that constitute the MOF and the nature of the IL segments (in terms of donor and acceptor number). Furthermore, by correctly matching the pore size of the MOF and the size of the ions from the IL, it is possible to modulate the electrochemical response of the system. Even so, some critical points on which focus the efforts to improve the performance of these hybrid materials exist, as detailed in Figure 8.

Future studies can be directed to:

1) Perform theoretical and computational studies. This type of studies, complementary to the experimental work, can be an essential guide in the design of hybrid electrolytes. These fundamental investigations play a key role in the understanding of the optimal MOF-IL combination that allows taking full advantage of the specific characteristics of each material.
2) Optimal selection of components. The chemical structure of the components is a critical factor, as it will govern the type and strength of the interactions. To complete this task, theoretical studies will become an important ally.
3) Study the critical structural factors in the electrochemical response of the electrolyte. Due to the early stage of the investigation of IL@MOF electrolytes, there is a great lack of studies on the influence of different factors such as particle size or the morphology, size and topology of the porosity on the electrochemical properties. This knowledge is essential to be able to advance in the development of these hybrid electrolytes.
4) Improve the values of ionic conductivity and transference number. Rational design of hybrid electrolytes, carefully selecting the components, trying to modulate the MOF-IL interactions in order to restrict the movement of the IL segments in the MOF pores and facilitating the mobility of lithium or sodium ions.
5) Analyze the stability of IL@MOF electrolytes in the highly reactive environment of the battery. Although there are some studies on the stability of IL@MOF materials in the plating/stripping processes, there are very few works on the use of IL@MOFs in half-cells. Moreover, it is essential to carry out post-mortem analyses of the materials in order to shed some light on how to avoid parasitic reactions that can reduce the life of the battery.
6) Solve mechanical problems. As mentioned above, MOFs provide rigidity to the electrolyte, but they are also brittle and can crack. The integration of IL@MOFs in polymer membranes is a strategy that can solve this drawback. The inclusion of MOFs helps the proper dispersion of the IL within the polymer. Furthermore, MOFs contribute to the decrease of crystallinity of the polymer, thus, increasing the ionic conductivity.
7) Delve into the process of mass production of hybrid IL@MOF electrolytes. It is necessary to develop and design membranes with the appropriate electrochemical and mechanical properties for their integration into batteries. It is interesting to explore scalable, environmentally sustainable and low-cost production processes, including additive manufacturing technologies.
